# Impact of Early-Commenced and Continued Sports Training on the Precuneus in Older Athletes

**DOI:** 10.3389/fnhum.2021.766935

**Published:** 2021-12-08

**Authors:** Masatoshi Yamashita, Maki Suzuki, Toshikazu Kawagoe, Kohei Asano, Masatoshi Futada, Ryusuke Nakai, Nobuhito Abe, Kaoru Sekiyama

**Affiliations:** ^1^Graduate School of Advanced Integrated Studies in Human Survivability, Kyoto University, Kyoto, Japan; ^2^Department of Behavioral Neurology and Neuropsychiatry, Osaka University United Graduate School of Child Development, Osaka, Japan; ^3^Faculty of Letters, Kumamoto University, Kumamoto, Japan; ^4^Liberal Arts Education Center, Kyushu Campuses, Tokai University, Kumamoto, Japan; ^5^Faculty of Child Care and Education, Osaka University of Comprehensive Children Education, Osaka, Japan; ^6^Kokoro Research Center, Kyoto University, Kyoto, Japan

**Keywords:** older athletes, precuneus, aging, early-commenced sports training, excessive training

## Abstract

Intervention studies on sedentary older adults have demonstrated that commencing physical exercise at an older age has a positive effect on brain structure. Although this suggests that older athletes with lifelong sports training have larger gray matter volume (GMV) in some brain regions compared to age-matched non-athletes, evidence in the literature is scarce. Moreover, it remains unclear whether a larger GMV is associated with training intensity or period of training in life. To address these gaps in the literature, we compared regional brain GMV between 24 older athletes (mean age, 71.4 years; age at the commencement of sports training, 31.2 years, continuous sports training, 40.0 years; current training time, 7.9 h/week) and 24 age-matched non-athletes (mean age, 71.0 years). The period of sports training and the current training time of the athletes were assessed. Both groups were evaluated for physical activity intensity as well as cognitive and motor performance. Although no group differences were noted in cognitive and motor performance, athletes reported higher physical activity intensity than non-athletes. Whole-brain structural analysis revealed a significantly larger GMV in several brain regions in athletes. Notably, the GMV of the precuneus in athletes was positively correlated with earlier commencement of sports training and training duration but was negatively correlated with current training time. Our findings demonstrate that early-commenced and continued sports training predicts structural maintenance of the precuneus in old age. Our results also suggest that excessive training time in old age may have a negative impact on the GMV of the precuneus; thereby delineating how the precuneus is associated with lifelong sports training in older athletes.

## Introduction

Magnetic resonance imaging (MRI) studies have revealed key structural characteristics of the aging brain. For example, the gray matter volume (GMV) of the frontal cortex, cingulate cortex, hippocampus, cerebellum, basal ganglia, and parietal lobe (e.g., supramarginal gyrus and precuneus) decreases with age (Raz et al., 2004, 2010; Ramanoël et al., 2018; Eyme et al., 2019; Hahm et al., 2019). Atrophy of these brain regions is associated with age-related decline in various behavioral measures of cognitive function (Seidler et al., 2010; Nyberg et al., 2012). Given the rise in aging-associated issues in current society, identifying lifestyle habits that effectively mitigate age-related cognitive decline and brain atrophy is essential.

Continued physical exercise is a candidate lifestyle factor in this regard, given its association with a reduced risk of dementia (Rovio et al., 2005; Larson et al., 2006; Tanaka et al., 2020) and mild cognitive impairment (Geda et al., 2010). Previous studies have reported that 6- to 12-month walking programs in sedentary older adults were associated with increased GMV of the hippocampus and frontal lobes, including the anterior cingulate cortex, supplementary motor area, and inferior frontal gyrus, compared to control participants who performed stretching as a non-aerobic exercise (Colcombe et al., 2006; Erickson et al., 2011). In addition, greater walking distance is associated with a larger GMV in the prefrontal and temporal regions, which reduces the risk of cognitive impairment (Erickson et al., 2010). These findings suggest that a physically active lifestyle predicts structural maintenance and related cognitive maintenance. Nevertheless, it remains unclear whether the effects of a physically active lifestyle are cross-sectionally evident in masters athletes relative to non-athletes.

Masters athletes are an excellent model of older adults who have participated in lifelong sports training and maintain high levels of motor skills. Several cross-sectional behavioral studies have reported higher levels of verbal memory, visuospatial processing, and attention in young (Lobjois et al., 2006; Tarumi et al., 2013) and older athletes (Lobjois et al., 2006; Tseng et al., 2013; Zhao et al., 2016) compared to that in age-matched non-athletes. These favorable characteristics of athletes may be underscored by physical fitness (e.g., muscle strength and cardiovascular endurance) as well as motor fitness (e.g., speed, balance, and fine motor coordination). The vigorous sports training of athletes may improve perceptual and motor processes associated with the mapping of sensation to action, which enables efficient spatial orientation and reactions to moving objects/persons. Moreover, metacognition in athletes is thought of as an essential component of self-regulation, proper strategies, and motor coordination monitoring to excel in sports (MacIntyre et al., 2014). Thus, sports training may lead to greater effects on cognitive function and brain structure compared to simple physical exercises, such as walking. Nevertheless, there is a paucity of studies on the effects of lifelong sports training on brain structure. One study reported that young athletes had larger GMV in the inferior parietal lobule, middle temporal gyrus, precentral gyrus, and frontal cortex compared to non-athletes (Fukuo et al., 2020). Another study reported larger GMV in the right parietal lobe (e.g., precuneus), occipital lobe, and cerebellum in a small group of older athletes compared to that in sedentary elderly individuals (Tseng et al., 2013). These findings suggest that the beneficial structural changes in the brains of athletes involve both motor-related and perception-related areas, highlighting the involvement of coordination between cognitive and motor processes when playing sports. Moreover, a study reported that current maximal oxygen consumption was positively correlated with the GMV of several brain structures, including the precuneus, frontal, and occipital lobes in older athletes (Tseng et al., 2013). However, these findings were based on a small sample. Further, the correlation was only observed between the current physical status of athletes and brain structure, and the study lacked information about the correlation between past athletic training and brain maintenance. Of note, previous studies have demonstrated that early-commenced physical training is associated with the strength of structural and functional connectivity in frontoparietal and default mode networks as well as related cognitive enhancement in middle-aged adults who have participated in regular sports training (Dik et al., 2003; Ferro et al., 2016; Ishihara et al., 2021). These findings suggest that early commencement of sports training may contribute to neural changes and related cognitive maintenance in later life and that the beneficial structural changes in the brains of athletes are affected not only by the amount of current physical activity (e.g., maximal oxygen consumption and current training time) but also by the length of experience, such as early-commenced and continued sports training. However, no study to date has identified the contribution of the length of sports experience to brain structure in older athletes.

This cross-sectional study aimed to address two research questions. First, we aimed to replicate the previously reported finding that the GMV in several brain regions was larger in older athletes compared to older non-athletes (Tseng et al., 2013) using a larger sample. Second, we aimed to determine whether the favorable structural changes in the brains of athletes, if any, were associated with the length of sports experience in life and/or the amount of current physical activity. To address these questions, we compared brain structure between older athletes and age-matched non-athletes. Based on the structural differences between groups, we further examined the associations between GMV in regions of interest (ROIs) and sports traits [age of commencement, years of training, current training time, and International Physical Activity Questionnaire (IPAQ) score]. We hypothesized that athletes would have increased the GMV in one or more brain regions (the precuneus, occipital lobe, and cerebellum) and that this increase would be correlated with the length of sports experience.

## Materials and Methods

### Participants

The Psychological Research Ethics Committee of Kumamoto University approved the protocol, and all study participants provided informed consent. In total, 49 older adults (25 athletes and 24 non-athletes, aged 65–79 years) participated in this study. Athletes were defined as those who had received sports training for at least 15 years, based on a previous study (Tseng et al., 2013). Athletes were recruited *via* the Kyoto City Silver Human Resources Center, Kyoto Kendo Hall, and personal connections associated with tennis, golf clubs, and dance schools. Non-athletes with less than 3 years of sports experience were recruited from the Kyoto City Silver Human Resources Center. All the participants were right-handed. The exclusion criteria were cognitive impairments [Table 1; Mini-Mental State Examination (MMSE) score ≥ 25], a history of neurological, cardiovascular, or psychiatric illness, and/or contraindications for MRI. One athlete was excluded due to physical deconditioning. The final analysis comprised 24 athletes (12 men and 12 women) and 24 non-athletes (12 men and 12 women).

**Table 1:** Demographics of athletes and non-athletes.

Total (*n* = 48)	Athletes (*n* = 24)	Non-athletes (*n* = 24)	*P*-value
Sex (male/female)	12/12	12/12	<1.000
Age (years)	71.4 (3.4)	71.0 (3.5)	<0.737
Education (years)	14.2 (2.2)	13.0 (2.3)	<0.077
Body height (cm)	160.1 (7.3)	159.9 (8.4)	<0.913
Body weight (kg)	56.9 (9.0)	58.1 (13.6)	<0.728
MMSE (score)	28.9 (1.2)	28.9 (0.9)	<0.891
IPAQ (score)	3479.9 (2133.1)	991.4 (1406.8)	<0.001

*The parameters are presented as mean (*SD*). *P*-values for age, education, body height and weight, MMSE, and IPAQ are from *t*-tests for group differences. *P*-value for sex ratio is from Chi-square test for group differences. MMSE, Mini-Mental State Examination; IPAQ, International Physical Activity Questionnaire; *SD*, standard deviation*.

The athletes had commenced sports training between the ages of 7 and 57 years (Table 2; mean = 31.2 years) and had been practicing sports for more than 19 years at the time the study was conducted (Table 2; mean = 40.0 years, range = 19–65 years). The current training time of the athletes was between 0.5 and 36.0 h per week (Table 2; mean = 7.9 h/week). The sports included tennis, kendo, golfing, dancing, cycling, and swimming. These sports, compared to simple physical exercises, such as walking and running, highlight the need for perceptual (e.g., visuospatial processing and attention shift) and motor (e.g., speed and balance) processing associated with sensation-to-action mapping.

**Table 2:** Sports training status in athletes.

Athletes (*n* = 24)	Mean (*SD*)	Range
Age of commencement	31.2 (15.0)	7−57
Years of training	40.0 (15.5)	19−65
Current training time (hours/week)	7.9 (7.2)	0.5−36

*SD, standard deviation*.

### Measures of Physical Activity

Physical activity intensity levels were assessed using the Japanese version of the IPAQ-Short Form, which contains seven questions (Craig et al., 2003; Inoue et al., 2009). In the IPAQ, participants were instructed to record their physical activity intensity levels (vigorous-intensity activity, moderate-intensity activity, walking, and sitting) over the previous 7 days.

### Behavioral Measurements

Cognitive and motor function and physical activity levels were measured and compared between athletes and non-athletes. Cognitive tests consisted of the Japanese versions of parts A and B of the trail-making test (TMT; Reitan and Wolfson, 1993) and logical memory-I and -II subtests of the Wechsler Memory Scale-Revised edition (WMS-R; Wechsler, 1997; Sugishita, 2000). Motor tests consisted of timed up and go (TUG; Podsiadlo and Richardson, 1991) and pegboard (Guo et al., 2020) tasks.

Parts A and B of the TMT were used to evaluate executive function. In TMT-A, participants were instructed to draw lines to sequentially connect 25 numbers in ascending order. In TMT-B, participants were instructed to draw lines alternately between numbers and letters (1, A, 2, B, etc.). The logical memory-I and -II subtests of the WMS-R were conducted to evaluate verbal memory. In the logical memory-I subtest, participants were instructed to immediately recall two stories sequentially. In the logical memory-II subtest, participants were instructed to recall the two stories 30 min later. The TUG and pegboard tasks were conducted to evaluate motor function. In the TUG test, participants were instructed to stand up from a standard chair, walk 3 m up and back, and sit down. In the pegboard task, participants were instructed to turn over 20 pegs (diameter × height = 0.5 × 3.5 cm) in 20 holes carved on a square board (length × width × thickness = 15 × 18 × 2 cm; SAKAI Medical Co., Ltd., Japan) using the right hand.

### Statistical Analysis of Behavioral Data

IPAQ scores and behavioral data were compared between athletes and non-athletes using a two-sample *t*-test for each of the seven measures. IBM-SPSS Statistics for Windows, version 25 (IBM corporation, Armonk, NY, USA) was used for statistical analysis. However, multiple *t*-tests may induce type I errors (overestimation of significant effects without correction) or type II errors (underestimation of significant effects under conservative correction, such as that using the Bonferroni method). The sampling method (e.g., permutation) can be used to estimate adjusted *P*-values while avoiding parametric assumptions about the joint distribution of the test statistics (Dudoit et al., 2003). A permutation test (Camargo et al., 2008) was conducted using MATLAB R2020a (The Mathworks Inc., United States) with a statistics and machine-learning toolbox. For each behavioral measure, all 48 observed samples (24 athletes and 24 non-athletes) were randomized together and resampled to obtain a dummy *t*-value. This procedure was repeated 10,000 times for each of the seven behavioral measures. A total of 70,000 *t*-values (10,000 resampling × 7 behavioral measures) were pooled and a unique permutation *t*-distribution was created to obtain a single adjusted α-level threshold (the top five percentile rank in the distribution).

### Image Acquisition

Scanning was performed using a 3T Siemens MAGNETOM Verio MR scanner (Siemens, Erlangen, Germany). The participants’ head was immobilized using a scanner head-coil (12 channels). High-resolution structural images were acquired using an axial T1-weighted magnetization-prepared rapid gradient-echo pulse sequence (*TR* = 2,250 ms; *TE* = 3.51 ms; *TI* = 900 ms; field of view = 256 × 256; matrix size = 256 × 256; voxel size = 1 × 1 × 1 mm; 208 slices).

### Image Preprocessing and Statistical Analysis of Structural Data

Voxel-based morphometry (VBM; Ashburner and Friston, 2000) was performed using the statistical parametric mapping software SPM12 (Wellcome Department of Cognitive Neurology, London) and MATLAB R2018a. Structural T1-weighted images were first segmented to separate the different types of tissues: gray matter, white matter, cerebrospinal fluid, soft tissue, and skull. The segmented images (gray matter and white matter) were then spatially normalized using the Diffeomorphic Anatomical Registration using Exponentiated Lie algebra (Ashburner, 2007). To preserve the absolute volume of gray matter, modulation was performed on normalized gray matter images by multiplying the Jacobian determinants derived from spatial normalization. Finally, the modulated gray matter images were smoothed with an 8-mm full-width at half-maximum Gaussian kernel.

For the VBM statistical analysis, we used threshold-free cluster enhancement (TFCE), which was introduced to enhance voxel-based analysis sensitivity, by applying 5,000 permutations (Smith method; Smith and Nichols, 2009; Salimi-Khorshidi et al., 2011; Nenadic et al., 2021). First, we tested the GMV differences between groups using a general linear model in the SPM software, with total brain volume defined as a nuisance variable. An explicit mask was used to exclude noisy voxels from the statistical analysis. Based on the TFCE analysis, the statistical thresholds were set at *P* < 0.001, uncorrected for multiple comparisons, and *P* < 0.05 family-wise error corrected for multiple comparisons. Thereafter, we identified the cluster location obtained from structural data using the anatomy toolbox in SPM12 (Eickhoff et al., 2005).

Next, the associations between sports traits and the GMVs in the ROIs were investigated. Based on the structural result differences between the groups, we used MarsBaR software (Brett et al., 2002) to extract spherical ROIs that centered on the local maximal peaks of the significant clusters (we used sub-clusters for overly large clusters) that were located within a 10-mm radius of the regions identified in the aforementioned analysis (Guo et al., 2020). Correlation analyses were conducted using the Pearson’s correlation coefficient. For these multiple coefficients, validation tests for correlation were performed using a permutation test in MATLAB R2020a. To examine the correlation for a given pair of variables (e.g., age at commencement of sports training and the GMV in the precuneus), a dummy coefficient was obtained by correlating the two variables randomly across participants. This procedure was repeated 10,000 times for each of the five correlations. A total of 50,000 dummy coefficients (10,000 resamplings × 5 correlations) were pooled and a unique permutation coefficient distribution was created to obtain a single adjusted α-level threshold (the top five percentile ranks in the distribution). For all analyses, statistical significance was set at *P* < 0.05.

## Results

### Demographics

Demographic data are presented in Table 1. No significant group differences were observed in age, years of education, body height and weight, and MMSE scores, indicating that the two groups were comparable, with the exception of sports traits. Welch’s *t*-test was used to analyze IPAQ scores due to heteroscedasticity. The results revealed a significant difference between groups (*t*_(41.69)_ = 4.66, *P* < 0.001, *d* = 1.32). This *t*-value was higher than the adjusted significance level threshold (*t*_(47)_ = 2.00) obtained in the permutation test, indicating that physical activity intensity levels were higher in athletes than in non-athletes.

### Behavioral Scores

Behavioral data are presented in Table 3 as mean ± standard deviation (*SD*). Based on a Student’s *t*-test, no significant between-group differences were identified in the TMT-A and -B, logical memory-I and -II subtests, TUG test, and pegboard task, indicating that behavioral performance was comparable between athletes and non-athletes in this study.

**Table 3:** Behavioral results in both groups.

Total (*n* = 48)	Athletes (*n* = 24)	Non-athletes (*n* = 24)	*P*-value
TMT-A (second)	37.9 (10.0)	36.5 (9.7)	0.625
TMT-B (second)	87.7 (18.7)	100.2 (43.4)	0.203
Logical memory-I (score)	18.3 (5.6)	18.8 (6.2)	0.811
Logical memory-II (score)	14.6 (5.9)	13.5 (5.0)	0.497
TUG (second)	6.0 (0.8)	6.3 (0.8)	0.265
Pegboard (second)	28.0 (4.2)	26.3 (4.2)	0.155

*The parameters are presented as mean (*SD*). *P*-values were obtained using *t*-tests for group differences. TMT, Trail-Making Test; Logical memory-I, immediate recall; Logical memory-II, delayed recall; TUG, time up and go; *SD*, standard deviation*.

### Sports Experience-Related Structural Changes

**Figure 1:**
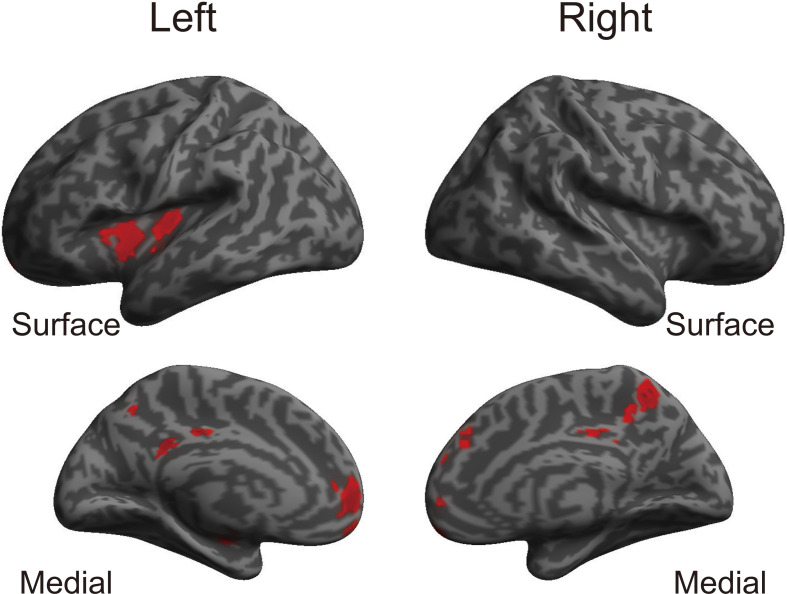
Brain regions where athletes showed larger GMVs than non-athletes. Using a TFCE analysis, the athletes showed increased GMVs in various brain regions compared to the non-athletes. Table 4 shows the brain regions where the athletes showed gray matter enlargement. TFCE, threshold-free cluster enhancement; GMV, gray matter volume.

**Figure 2:**
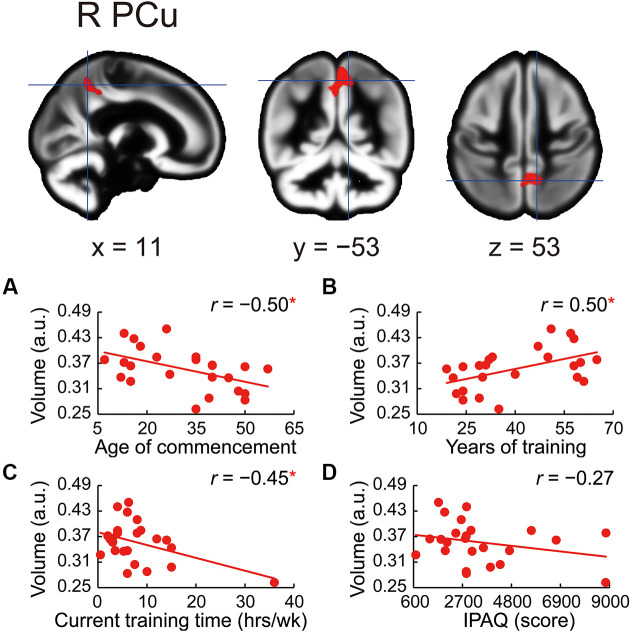
Correlations between precuneus volume and sports traits in the athletes. GMV in the right precuneus was larger in athletes than in non-athletes. Using ROIs in the precuneus, **(A)** age at the commencement of sports training was negatively correlated with GMV of the precuneus in athletes. **(B)** GMV of the precuneus was positively correlated with the number of years of sports training in athletes. **(C)** GMV of the precuneus was negatively correlated with current training time in athletes. **(D)** No significant correlation between GMV of the precuneus and IPAQ scores in athletes was noted. *P < 0.05, GMV, gray matter volume; ROI, region of interest; R PCu, right precuneus; a.u., arbitrary units; hrs/wk, hours per week; IPAQ, International Physical Activity Questionnaire.

The whole-brain voxel-based gray matter analyses revealed significantly larger GMVs in the left middle cingulate gyrus, left posterior cingulate gyrus, right precuneus, left superior frontal gyrus, right frontal pole, left posterior and anterior insula, and right superior frontal gyrus (Table 4 and Figure 1). GMVs of ROIs in these regions in athletes were extracted, and the associations between GMV and sports traits were investigated (Figure 2). Age of commencement and continuous sports training were correlated with the GMV of the precuneus in athletes (age of commencement: *r* = −0.50, *P* = 0.014; years of training: *r* = 0.50, *P* = 0.013). In addition, GMV of the precuneus was negatively correlated with current training time (*r* = −0.45, *P* = 0.027). These Pearson’s *r*-values were higher in absolute value than the adjusted significance level threshold (|*r*| = 0.40) obtained in the permutation test. In contrast, no significant correlation between the GMV of the precuneus and IPAQ scores was observed in athletes (*r* = −0.27, *P* = 0.20) or in non-athletes (*r* = −0.049, *P* = 0.82). GMV of the precuneus was greater in athletes with earlier training commencement and longer training duration, highlighting the influence of lifelong sports training.

**Table 4:** Brain areas with significantly larger gray matter volume in athletes than in non-athletes (TFCE analysis, *P* < 0.05, FWE corrected).

Location	MNI coordinates			*P*-value	Cluster size
	*x*	*y*	*z*		
Left middle cingulate gyrus	−2	−23	42	0.030	1,860
Left posterior cingulate gyrus	0	−39	30	0.031	
Right precuneus	11	−53	53	0.038	
Left superior frontal gyrus	−12	54	2	0.034	1,740
Right frontal pole	6	62	−18	0.038	
Left posterior insula	−35	−17	8	0.037	1,576
Left anterior insula	−42	−2	−2	0.040	
Right superior frontal gyrus	2	47	26	0.046	352

*For overly large clusters, we also showed sub-clusters. TFCE, threshold-free cluster enhancement; FWE, family-wise error; MNI, Montreal Neurological Institute*.

## Discussion

This study compared the brain structure in older athletes with that in age-matched non-athletes. The correlation between GMV in athletes and sports traits was also investigated. Although no significant differences in cognitive and motor performance were observed between athletes and non-athletes, athletes demonstrated higher physical activity intensity levels, as measured using the IPAQ, compared to that of non-athletes. Compared to the non-athletes, the GMVs were larger in several of the brain regions in the athletes. Notably, the GMV of the precuneus was correlated with the early commencement of sports training and training duration. In contrast, the GMV of the precuneus in athletes was negatively correlated with current training time. IPAQ scores, which reflect the total intensity levels of multiple types of physical activity, were not correlated with the GMV of the precuneus. These findings suggest that early-commenced and continued sports training, rather than the amount of current physical activity, predict the structural integrity of the precuneus in old age. Additionally, the negative correlation between GMV of the precuneus and current training time implies that excessive physical training in old age may be detrimental. Since the aim of this study was to clarify which essential brain regions are associated with lifelong sports training, the present discussion is restricted to our findings regarding the correlation between sports experience traits and the precuneus in older athletes.

In contrast to previous studies reporting that young and older athletes exhibit better verbal memory and executive function performance (Tarumi et al., 2013; Tseng et al., 2013), no significant differences in WMS-R and TMT task performance were observed between athletes and non-athletes in this study. A possible reason for this discrepancy is the involvement of other lifestyle factors such as frequency of cognitive activity and social interaction; these factors were not controlled between the groups, which may have weakened the effect of sports. In this study, participants in the control group were recruited from the Kyoto City Silver Human Resources Center, i.e., they were working part-time. Therefore, they may have been more active than sedentary older adults. Another possible reason for the discrepant findings is the age difference between the groups. In the study by Tseng et al. (2013), the mean ages of athletes and non-athletes were 72.4 and 74.6 years old, respectively. In contrast, the mean ages of athletes and non-athletes in the current study were 71.4 and 71.0 years, respectively. Thus, in the study by Tseng et al. (2013), compared to the athlete group, the non-athlete group may have included more participants that were older than 75 years. In this regard, an advanced decline in executive function tends to occur at this age (Suzuki et al., 2018).

Although no significant differences in behavioral performance were observed between athletes and non-athletes in this study, the GMVs of the various brain regions were larger in the athletes than in the non-athletes. Of note, the GMV of the precuneus was significantly associated with sports experience traits in athletes. Specifically, larger GMV of the precuneus in athletes was associated with earlier commencement of sports training and training duration. These findings suggest that lifelong sports training may result in beneficial structural changes in the brain. These findings are consistent with previous studies. For instance, our results extend the findings from a previous study of young athletes (Fukuo et al., 2020) to include older populations. This consistency suggests that the precuneus is an essential region affected by sports training. Further, the GMV of the precuneus in older athletes was positively correlated with early commencement and duration of sports training. This extends a previous finding in middle-aged athletes (Eyme et al., 2019) to the older population. In agreement with the well-established aging-related deterioration of the precuneus (Eyme et al., 2019; Hahm et al., 2019), our findings validate the analogous effects of sports in older athletes (≥65 years). The current results also highlight the benefits of commencing sports training in early life and continuing training into old age to maintain the structural integrity of the precuneus.

The precuneus is involved in self-awareness, metacognitive efficiency, and attentional shift (Nagahama et al., 1999; Gilboa et al., 2004; Ye et al., 2019) and plays an important role in visuospatial imagery for body movement control (Ogiso et al., 2000; Suchan et al., 2002; Tian et al., 2019). Malouin et al. (2003) reported activation of the precuneus in imagery tasks of walking with obstacles through a virtual environment, suggesting the involvement of the precuneus in the efficient predictive adaptation of postural control, motor coordination, spatial orientation, and reaction to moving objects/persons. Moreover, the precuneus has been implicated in the processing capacity of visuospatial information in athletes (Guo et al., 2017). The accumulation of training in motor skills may strongly influence the precuneus in old age, presumably because visuospatial processing, self-awareness, metacognition, and attentional shift are essential for playing sports. The increased GMV of the precuneus and its association with the length of sports experience observed in our study suggests that athletes may maintain motor skills involving high cognitive demand at an old age.

In addition, the current findings highlight the potential effects of sports on the default mode network. The precuneus and posterior cingulate cortex comprise the functional core of the default mode network (Wang et al., 2006; Utevsky et al., 2014). Connectivity in the default mode network at rest has been reported to decrease with age (Damoiseaux et al., 2008; Ferreira and Busatto, 2013), and this suppression may be associated with a reduction in cognitive control (Dik et al., 2003; Ferro et al., 2016; Ishihara et al., 2021). In contrast, early-commenced motor training is associated with the strength of structural connectivity, including that in the default mode network, in middle-aged adults (Dik et al., 2003; Ferro et al., 2016; Ishihara et al., 2021). In our study, a positive change in the GMV of the precuneus was observed in athletes due to long-term sports experience. Future research should investigate the relationship between the maintenance of the GMV of the precuneus and connectivity in the default mode network.

Previous studies have reported that a higher amount of physical activity (maximal oxygen consumption) is associated with a larger GMV of the precuneus (Prakash et al., 2010; Tseng et al., 2013; Boots et al., 2015; Castells-Sánchez et al., 2021). In contrast, the present study revealed a negative association between the GMV and current training time, suggesting that the GMV of the precuneus in athletes decreased with prolonged training time. A relevant concept in this regard is the U-shaped function of the relationship between physical exercise intensity and cardiovascular state, whereby moderate physical exercise is better than no exercise, but vigorous physical exercise is associated with adverse cardiovascular events (Merghani et al., 2016). The latter may be due to the metabolic activation of the tryptophan-kynurenine pathway in the brain as a result of prolonged physical activity and intense physical exercise, which can lead to central fatigue (Strasser et al., 2016; Yamashita, 2020). These neuroactive metabolites are associated with cognitive decline and brain deterioration (Yamashita and Yamamoto, 2017; Yamashita, 2020). Moreover, several studies have demonstrated that central fatigue may influence the reduction in the GMV in the cortical and subcortical regions (de Lange et al., 2005; Riccitelli et al., 2011; Arm et al., 2019). Higher concentrations of tryptophan and kynurenine have been observed in younger and older adults during prolonged physical exercise (Melancon et al., 2012; Strasser et al., 2016; Trepci et al., 2020). Prolonged physical activity and its high load in older athletes may be associated with structural deterioration due to central fatigue. If the relationship between the amount of current physical exercise and GMV of the precuneus is underpinned by a U-shaped function, moderate sports training may have beneficial effects on brain health. To interpret previous reports (Prakash et al., 2010; Tseng et al., 2013; Boots et al., 2015; Castells-Sánchez et al., 2021) and the current findings uniformly in accordance with a U-shaped function, maximal oxygen consumption may be a useful physical measure to distinguish lower amounts of physical exercise, whereas current training time may indicate a higher amount of exercise.

Our study has several limitations. First, our study was cross-sectional; thus, causal relationships between sports training and changes in GMV in athletes could not be drawn conclusively. Second, the present study was unable to recruit a sufficient number of athletes for each sport to investigate the effects of sports type on brain structure and cognitive function. Therefore, the effects of sports type on the current results remain unclear. Third, the present study did not observe significant differences in cognitive and motor performance between athletes and non-athletes, presumably because extraneous variables may have weakened the effects of sports training. Therefore, future research should control for lifestyle factors, such as the frequency of cognitive activity and social interaction.

## Conclusion

Compared to age-matched non-athletes, older athletes exhibited beneficial structural changes in the precuneus, and the GMV of the precuneus was significantly associated with sports traits. Although no significant group differences in cognitive and motor performance were observed between athletes and non-athletes, athletes exhibited lower levels of brain atrophy in the precuneus. Moreover, early-commenced and continued sports training were associated with a larger GMV in the precuneus. However, excessive sports training may have negative effects on the GMV in the precuneus. The present findings shed new light on the effects of lifelong sports training on the maintenance of brain health in old age.

## Data Availability Statement

The raw data supporting the conclusions of this article will be made available by the authors, without undue reservation.

## Ethics Statement

The studies involving human participants were reviewed and approved by The Psychological Research Ethics Committee of Kumamoto University. The patients/participants provided their written informed consent to participate in this study.

## Author Contributions

KS and MS designed the research. MS, TK, and MF performed the research. MY, MS, TK, KA, MF, RN, NA, and KS contributed experimental materials and tools. MY and MS analyzed the data. MY and KS wrote the original manuscript. MY, MS, TK, KA, MF, RN, NA, and KS revised the manuscript. All authors contributed to the article and approved the submitted version.

## Conflict of Interest

The authors declare that the research was conducted in the absence of any commercial or financial relationships that could be construed as a potential conflict of interest.

## Publisher’s Note

All claims expressed in this article are solely those of the authors and do not necessarily represent those of their affiliated organizations, or those of the publisher, the editors and the reviewers. Any product that may be evaluated in this article, or claim that may be made by its manufacturer, is not guaranteed or endorsed by the publisher.
